# Editorial: Neurocomputational models of language processing

**DOI:** 10.3389/fnhum.2024.1524366

**Published:** 2024-12-02

**Authors:** Markus J. Hofmann, Ya-Ning Chang, Harm Brouwer, Michael Zock

**Affiliations:** ^1^Department of Psychology, University of Wuppertal, Wuppertal, Germany; ^2^Miin Wu School of Computing, National Cheng Kung University, Tainan, Taiwan; ^3^Department of Cognitive Science and Artificial Intelligence, Tilburg University, Tilburg, Netherlands; ^4^UMR7020 Laboratoire d'Informatique et Systèmes (LIS), Marseille, France

**Keywords:** neurocognitive model, connectionist, expert system, machine learning, language model, brain-computer interface, mathematical modeling

The ability to predict and explain is fundamental to any field aspiring to be considered a science. To move closer toward this goal, we invited submissions on neurocomputational models of language processing. Unlike linguistic models, which are primarily theoretical, these new models are testable by behavioral and neural data. Therefore, they can claim to be part of natural sciences.

Our objective was to bridge or at least narrow the gap between computational models, language *processed by machines*, and language *processed by humans*. While the former yield practical results, the latter provide behavioral and neural data that are invaluable for gaining insights into the strategies used by the human mind and brain to process language. In other words, they may shed some light on the relationship between cognitive processes involved in language processing in reception or production tasks and the functioning of the human brain.

Since the workings of the human neural system are still not well-understood, creating models that can measure behavior at the neural level remains a challenge. This is not surprising, given that the field of the neurocognitive sciences is still relatively young.

To narrow the gap, we have invited researchers to address some of the issues mentioned in our call for papers. We included seven papers in this Research Topic.

Huang et al. proposed a neural dipole model to simulate how different brain regions interact. Although such interactions are often explored in fMRI studies, the authors examined this in EEG data. They also collected eye movement data during reading aloud to examine the eye-voice span. Their findings indicated that the time lag between eye fixations and voice onset decreased when discourse was coherent but remained constant in the opposite case. A neural network involved in reading, spanning from the superior parietal cortex to the frontal lobe, exhibited stronger connectivity when participants read incoherent sentences. This nicely illustrates the role of coherence, cohesion, semantics and context as facilitating factors for reading.

Brain-computer interfaces (BCIs) represent one of the most promising applications of neurocomputational models, because brain activation is here taken to control technical devices. Their current effectiveness, however, remains quite limited. Herbert proposes a roadmap to better attune BCIs to linguistic and cultural differences. She argues that interindividual variations in perception, action, cognition, language, and emotion must be considered in BCIs, and that language training could enhance their performance. Her work aligns well with another Research Topic on language, culture and the brain.

While Herbert's goal of refining computational architectures was grounded in linguistic theories, Blache took a different approach. He reflected on classical linguistic theories and explored how they can be reconciled with recent developments within the memory unification and control theory, as well as with the predictive coding framework. His formal approach could serve as an interpretable alternative to self-learning systems, once it is implemented as an algorithmic model. Even if such a model may not predict data as effectively as machine learning models, it may offer a framework that can be experimentally tested. We believe that the strategy used in physics, which divides labor between experimental and theoretical scientists, may also be beneficial for researchers in linguistics and the social sciences.

Though algorithmic models provide more formal tools for expressing theoretical ideas, physics, as the foundational natural science, often relies on mathematical models. While neural models excel at prediction, mathematical models – such as regression models – can offer better explanatory power and computational efficiency. Heitmeier et al. investigated the performance of such models during Dutch visual word recognition and Mandarin auditory lexical decision tasks. They also simulated the development of child-directed speech, considering how word frequency changes over time, noting that certain words might be frequent in early learning but rarely used later.

There are many models available for various applications. Among the most successful are connectionist models. Chang et al. trained a variant of the triangle model with phonology-focused and meaning-focused instructions, as well as with an instruction balancing phonology and meaning. They found that a semantic reading instruction led to greater reliance on meaning rather than phonology. It also resulted in more phonological errors, possibly due to individually developed reading styles. Their work ties into previous behavioral and neuroimaging studies, highlighting how computational models can simulate individual differences in reading strategies, especially for beginning readers.

Kohonen self-organizing maps, another type of model rooted in early connectionist ideas, were used by Weitin et al. in their study of literature processing. Participants read original passages from Harry Potter, well-written fan fiction, or poorly written fiction, dubbed “bad fiction.” The poorly written material elicited the lowest EEG activity, suggesting that high-quality literature resonates more strongly in the brain. Furthermore, fans of Harry Potter exhibited heightened brain activity in the theta and alpha bands. Weitin et al.'s team demonstrated that their Kohonen map could reliably classify original text from fan fiction and bad fiction based on EEG data.

Finally, Britton et al. investigated discourse connectives such as “even so” and their role in reversing expectations between sentences. Their study, conducted with Italian and Chinese speakers, confirmed that expectation reversal increases the plausibility of contradictory sentences in Italian but not in Chinese. They also found that deep neural networks struggle to recognize connectives as cues for expectation reversal, especially in morphologically rich languages like Italian, where tokenization often results in sub-word tokens that hinder fluent processing.

There are still many mysteries to be unraveled on how the brain generates the human mind during language processing. After seven decades of progress in connectionist models, however, the recent Nobel Prize in Physics awarded to Hinton and Hopfield serves as a testament to the value of ongoing theoretical development, even when the path ahead appears complex and is often overlooked or misunderstood. Let's use this example as inspiration to keep moving forward and trust our intuitions, much as Hinton did for many decades even when expert system technology seemed to have won the race in AI research.

